# The role of actin isoforms in somatic embryogenesis in Norway spruce

**DOI:** 10.1186/1471-2229-10-89

**Published:** 2010-05-17

**Authors:** Kateřina Schwarzerová, Zuzana Vondráková, Lukáš Fischer, Petra Boříková, Erica Bellinvia, Kateřina Eliášová, Lenka Havelková, Jindřiška Fišerová, Martin Vágner, Zdeněk Opatrný

**Affiliations:** 1 Charles University in Prague, Faculty of Science, Department of Plant Physiology, Viničná 5, CZ 12844 Prague 2, Czech Republic; 2 Institute of Experimental Botany v.v.i., Academy of Sciences of the Czech Republic, Rozvojová 236, CZ 16502 Prague 6, Czech Republic; 3 School of Biological and Biomedical Sciences, Durham University, South Road, DH1 3LE, UK

## Abstract

**Background:**

Somatic embryogenesis in spruce is a process of high importance for biotechnology, yet it comprises of orchestrated series of events whose cellular and molecular details are not well understood. In this study, we examined the role of actin cytoskeleton during somatic embryogenesis in Norway spruce line AFO 541 by means of anti-actin drugs.

**Results:**

Application of low doses (50-100 nM) of latrunculin B (Lat B) during the maturation of somatic embryos predominantly killed suspensor cells while leaving the cells in meristematic centres alive, indicating differential sensitivity of actin in the two cell types. The treatment resulted in faster development of more advanced embryos into mature somatic embryos and elimination of insufficiently developed ones. In searching for the cause of the differential actin sensitivity of the two cell types, we analysed the composition of actin isoforms in the culture and isolated four spruce actin genes. Analysis of their expression during embryo maturation revealed that one actin isoform was expressed constitutively in both cell types, whereas three actin isoforms were expressed predominantly in suspensor cells and their expression declined during the maturation. The expression decline was greatly enhanced by Lat B treatment. Sequence analysis revealed amino-acid substitutions in the Lat B-binding site in one of the suspensor-specific actin isoforms, which may result in a different binding affinity for Lat B.

**Conclusions:**

We show that manipulating actin in specific cell types in somatic embryos using Lat B treatment accelerated and even synchronized the development of somatic embryos and may be of practical use in biotechnology.

## Background

Embryogenesis in multicellular organisms comprises orchestrated processes that determine the spatio-temporal cell positioning in the developing embryo. In higher plants, which lack active cell movement, the structure and position of individual tissues are determined solely by the orientation of cell division, cell growth and developmentally regulated programmed cell death (PCD). Various cytoskeletal structures have been repeatedly demonstrated to play a key role in all these processes.

Actin is a ubiquitous component of all eukaryotic cells. In plants, actin cytoskeleton participates in the definition of cell polarity and orientation of cell division, cell elongation, cell wall development, transport processes, positioning of membrane receptors (for reviews see [1]), and in PCD [2,3]. To fulfil such a variety of specific roles, the arrangement and dynamics of actin cytoskeleton must be precisely regulated, including the composition of actin isoforms as well as the composition and activity of associated proteins. Multiple genes encoding actin isoforms, which are more or less specifically expressed in particular tissues or organs, have appeared during plant evolution [4-7]. Studies of actin gene expression in *Arabidopsis thaliana *have led to their subdivision into vegetative and reproductive classes, which probably diverged early in land plant evolution [6-8]. The apparent functional non-equivalence of actin isoforms has been documented not only by their different and characteristic developmental expression profiles but also by genetic manipulation. In *Arabidopsis*, ectopic expression of a reproductive actin gene under the control of a vegetative actin promoter severely altered organ morphology and caused dwarfism [9]. On the other hand, some actins can complement each other in their biological role [10] although elimination or mutation of other actin genes can lead to fatal phenotypes.

Zygotic embryogenesis represents a complex developmental process that includes all the morphogenic activities mentioned above - cell division, cell growth and differentiation as well as PCD. Analyses of early zygotic embryogenesis are technically complicated by the fact that zygotic embryos normally develop under several tissue layers *in planta*. Therefore, an alternative experimental system of somatic embryogenesis (SE) *in vitro *is commonly used for this purpose [11-13]. SE of conifers has also attracted much attention in recent years, being the promising technology for clonal propagation and breeding programs in forestry [14,15]. The principal stages of conifer somatic embryo development have been characterised according to their similarity to corresponding developmental stages in zygotic embryos [16,17]. Generally, conifer SE lines consist of two distinct cell types: isodiametric cells of meristematic centres ("embryonal heads") containing dense cytoplasm and long, vacuolated suspensor cells [16].

The role of the cytoskeleton in the formation of somatic embryos in *Picea abies *was demonstrated by Smertenko *et al*. [18]. The authors showed that the cytoskeleton organization differed in meristematic and suspensor cells and that the microtubule-associated protein MAP65 played a role in the onset of early embryo development. They also observed that the application of anti-actin drugs latrunculin B (Lat B) and cytochalasin D (Cyt D) prevented embryo formation, most probably *via *the inhibition of developmentally regulated PCD in suspensor cells [2]. However, they found no pronounced differences in Lat B and Cyt D effects and no positive impact on the SE process was reported. Till now, more detailed cytological and molecular analysis of the behaviour of both microtubular and actin cytoskeleton during the embryogenic process in SE lines was missing.

Based on our previous studies on the mechanisms of SE regulations in conifers [17] we hypothesised that actin cytoskeleton may play a role in developmental defects in particular SE conifer lines. In this study, using Norway spruce embryogenic line AFO 541 we found three actin isoforms that were expressed in a cell type-specific manner. Application of the anti-actin drug latrunculin B (Lat B) to embryogenic cells in order to study the effect of actin depolymerization on somatic embryogenesis revealed that the treatment promoted the development of high-quality embryos while suppressing the formation of non-mature embryos. The mechanism of this effect is explained by selective elimination of suspensors due to higher actin sensitivity towards Lat B in suspensor cells relative to meristematic cells.

## Results

### Embryogenesis in Norway spruce line AFO 541 cultured on proliferation and maturation media

**Figure 1:**
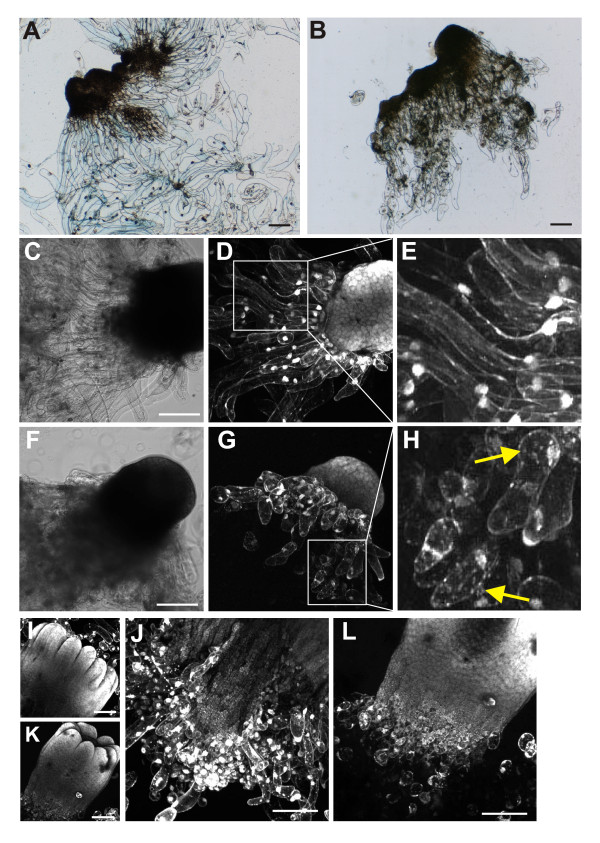
**Phenotype and viability of Norway spruce somatic embryos during maturation on media containing latrunculin B**. Vital staining of ESM with trypan blue (A, B) or with fluorescein diacetate (D, E, G, H, I-L). (A, B) ESM maturing for one week on a control medium (A) or a medium supplemented with 50 nM Lat B (B). Suspensors in controls consist of long viable cells (A), whereas most suspensor cells are dead in embryos maturing in the presence of Lat B (B). (C-H) ESM maturing for two weeks on a control medium (C-E) or a medium containing 50 nM Lat B (F-H). Long viable cells form long suspensors in the controls (D, E), whereas suspensors in the presence of Lat B consist of few short cells (G, H). Fluorescein diacetate staining revealed numerous granules in the cytoplasm of these short cells (arrows in H), indicating dying cells. (I-L) Three weeks of maturation. Cotyledons have developed in both treatments (I, K). A high degree of suspensor cell degradation is visible in the controls (J) and only few suspensor cells remain in embryos maturing in the presence of 50 nM Lat B (L). Scale bars, 200 μm.

Embryogenic suspensor mass (ESM) cultured continuously on proliferation media consisted of single early somatic embryos, free suspensor cells and polyembryogenic complexes. Polyembryogenic complexes and somatic embryos consisted of long suspensor cells connected with meristematic centres. During the proliferation phase, new embryogenic structures emerged and older structures were degraded. Further development of ESM to mature embryos was induced by transfer onto maturation media devoid of auxin and cytokinins but supplemented with abscisic acid. The meristematic centres gradually increased and individual embryos were released from polyembryogenic complexes. The maturation process was somewhat asynchronous so that the cultures consisted of embryos at various stages of development and quality. The suspensors were gradually degraded during the first three weeks of maturation. Their cytoplasm became granular and the cells eventually died, so that the frequency of suspensor cells connected to individual meristematic centres decreased (Figure 1A, C-E, I, J). After four weeks of maturation, only the largest embryos with distinct cotyledons were fully developed. Mature cotyledonary embryos generally lacked suspensors.

### Latrunculin B accelerates the maturation of somatic embryos and positively selects well-developed embryos

To examine the role of actin cytoskeleton in the process of somatic embryogenesis, we applied the actin depolymerising drug latrunculin B (Lat B) either to proliferation or maturation cultures. The extent and the speed of the response were dose-dependent. In general, broad concentration scale of Lat B (1, 10, 25, 50, 100, 200 nM) was tested. In the proliferating cultures, Lat B at the concentration of 1 nM had no effect, but 10 nM Lat B induced cell death of suspensor cells. Progressive death of the suspensors was followed by disintegration of the meristematic centres and subsequent death of their cells. At 100-200 nM concentrations, Lat B was clearly lethal (data not shown).

When applied to maturation cultures, 50-100 nM Lat B caused disintegration of suspensors already after one week of exposure to the drug (Figure 1B, compare with control in Figure 1A). After two weeks, the suspensor cells of untreated culture were large, elongated, and showed high viability (Figure 1C-E), whereas the suspensor cells in the Lat B treatment clearly showed decreased viability (Figure 1F, G). The surviving suspensor cells were short, with granular cytoplasm indicative of cell death (Figure 1G, H). Interestingly, the meristematic cells of the embryos were not directly influenced by the drug; they remained fully viable and underwent further development (Figure 1D, G). After three weeks, embryos with differentiated cotyledons were seen in both controls and the Lat B treatments, and the cells remained viable (Figure 1I-L). In contrast to the controls, the suspensors of embryos treated with Lat B were almost completely disintegrated (Figure 1L).

**Figure 2:**
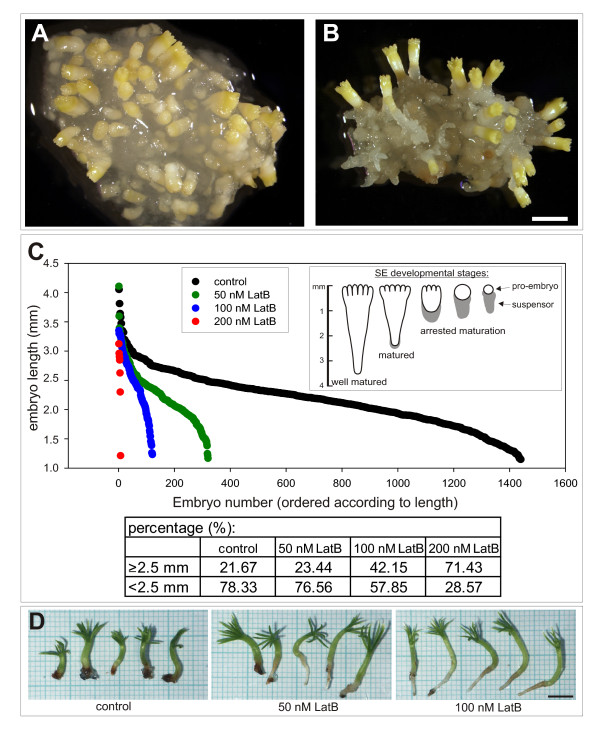
**Effect of latrunculin B on the yield and quality of somatic embryos**. (A, B) ESM after four weeks of maturation. Numerous embryos at various stages of development formed in the controls (A), whereas fewer uniform embryos at advanced developmental stage formed in the presence of 100 nM Lat B (B). (C) Comparison between the number of embryos and their length after four weeks of maturation in the controls or on media containing various Lat B concentrations. Each point in the graph corresponds to one embryo and all embryos in each variant were arranged descending by size. The table shows percentage representation of embryos longer and shorter than 2.5 mm from the experiment shown in the graph (see Methods for details on the yield evaluation). The embryo length was established as a simple criterion of embryo maturity (see inset), where the embryo length 2.5 mm was considered as the minimal length for matured embryos. (D) Germination of somatic embryos for 2 weeks. Embryos that had matured in the presence of Lat B had a high ability to germinate. Scale bars are 2 mm (A, B) or 5 mm (D).

By the end of four weeks of maturation, the control cultures consisted of a mixture of embryos at various stages of differentiation (Figure 2A, C). Surprisingly, development of embryos in the presence of Lat B was slightly faster compared with untreated controls (Figure 2A, B). Though the Lat B-treated culture produced significantly fewer embryos, the proportion of well-developed/matured ones increased by nearly twice under 100 nm Lat B (table in Figure 2C), when we used the embryo length as a simple substitutional criterion of embryo maturity (embryos above 2.5 mm were considered as matured ones; see inset in Fig. 2C and details in Methods). The effect of 50 nM LatB was less prominent in both aspects - the total number of embryos was less reduced, but simultaneously the proportion of matured embryos increased only slightly. The embryos that matured in the presence of Lat B showed no morphological abnormalities, were fully viable and germinated normally (Figure 2D).

Various time points of Lat B addition to maturating embryos were tested; the addition at the beginning of maturation was the most effective, applications in the second week of maturation were less effective and no effect on the embryo yield and maturity was observed when Lat B was applied at the third and forth week of maturation (data not shown).

### Actin cytoskeleton in suspensor cells is distinctly sensitive to latrunculin B

**Figure 3:**
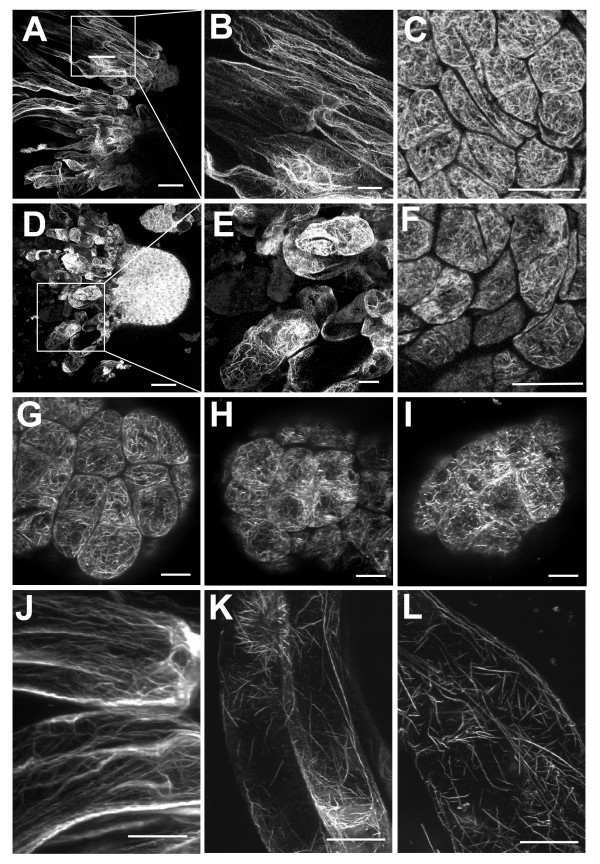
**Confocal images of actin cytoskeleton stained with rhodamine-phalloidin**. ESM after two weeks of maturation on control media (A-C) or media supplemented with 50 nM Lat B (D-F). Images show actin in polyembryogenic complexes (A) and in separated embryos (D) consisting of suspensor cells (B, E) and meristematic centers (C, F). (G-L) ESM after four days maturation on control media followed by a short (1 hour) treatment with Lat B. Actin in meristematic cells (G) and suspensor cells (J) in the controls. Actin in meristematic cells following a 1-hour treatment with 50 nM (H) or 200 nM Lat B (I). Actin in suspensor cells following a 1-hour treatment with 50 nM (K) or 200 nM Lat B (L). Scale bars are 100 μm (A, D) and 30 μm (B, C, E, F, G-L).

Actin cytoskeleton in meristematic cells normally consisted of dense, randomly oriented actin network (Figure 3C, G) whereas suspensor cells showed long, thick actin cables oriented longitudinally to the cell axis accompanied by transversely oriented thinner actin filaments (Figure 3B, J). The organization of actin cytoskeleton remained unchanged in ESM cultivated on proliferation or maturation media for 4 or 10 days, and no specific reorganization of actin cytoskeleton was observed at the onset of maturation. During maturation in the presence of 50 nM Lat B, actin cytoskeleton in the suspensor cells was largely degraded, forming shorter randomly oriented polymers (Figure 3E), although the effect was apparently milder in the meristematic cells. Though the actin filaments appeared shorter in the presence of Lat B than those in the controls (compare Figure 3C and 3F), they still formed a dense network (Figure 3F). Treatment with the solvent DMSO alone had no detectable effect on actin organization (data not shown).

To analyze the immediate effects of Lat B on actin cytoskeleton, we cultivated embryos on normal maturation media for four days and then applied various concentrations of Lat B for one hour. The short treatment using 50 nM Lat B induced only minor changes in actin organization in meristematic cells, leaving a control-like dense network of filaments (Figure 3H, compare with control in Figure 3G). However, the same treatment resulted in severe fragmentation of the long actin cables in the suspensor cells (Figure 3K, compare with control 3J). Treatment with 200 nM Lat B dramatically disrupted actin organisation in both the suspensor and the meristematic cells. Actin cables in suspensor cells were almost completely fragmented into short, randomly oriented rods (Figure 3L), and actin filaments in meristematic cells were fragmented into shorter bundles (Figure 3I) or even fully depolymerized.

### Multiple actin isoforms are expressed in the Norway spruce line AFO 541

**Figure 4:**
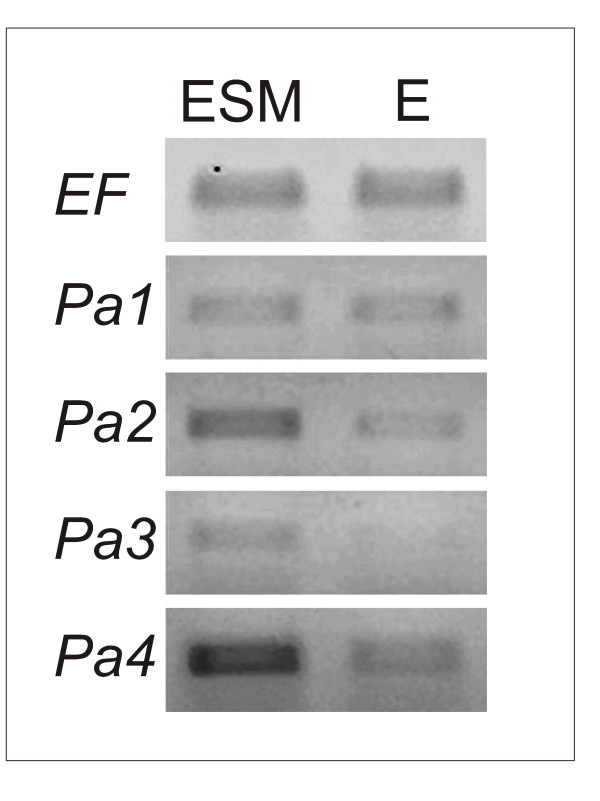
**Expression of actin isoforms in control ESM and isolated embryos determined by semiquantitative RT-PCR**. Transcript levels in non-fractionated ESM culture (ESM) and in isolated embryos (E) after two weeks of maturation on control media. *EF*, gene for elongation factor ef1α (internal standard); *Pa1-4*, *Picea abies *actin isoforms 1-4. For details see Material and Methods and Results.

Because our results showed that the sensitivity of actin cytoskeleton to Lat B is different between meristematic and suspensor cells in the embryogenic line, we embarked on analyzing the expression of actin genes as a possible cause. The number of spruce gene sequences in public databases is very limited, therefore we first searched for additional actin genes using RT-PCR and either degenerate primers or specific primers designed according to actin-encoding ESTs from *Pinus taeda*. In this way four new actin genes, three complete and one truncated, were isolated from the Norway spruce (*Picea abies*) line AFO 541 and named *Pa1*-*4 *(NCBI Accession numbers FJ869868-FJ869871). We then analyzed the expression levels of these four actin isoforms by semiquantitative RT-PCR using either RNA prepared from maturing ESM that contained both meristematic centres and suspensor cells, or RNA prepared from a purified fraction that contained meristematic centres alone. The expression pattern revealed that the levels of isoforms *Pa2*, *Pa3 *and *Pa4 *in the non-fractionated ESM were distinctly higher than those in the purified fraction of meristematic centres (Figure 4). This result clearly indicates that the expression of the isoforms *Pa2*, *3 *and *4 *predominated in suspensor cells, whereas the isoform *Pa1 *was expressed more or less equally in both cell types.

**Figure 5:**
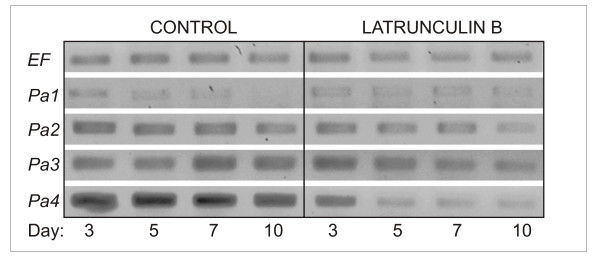
**Expression of actin isoforms during maturation in the presence of Lat B as determined by semiquantitative RT-PCR**. Transcript levels in non-fractionated ESM cultures were monitored during 10-days maturation on control media or media with the addition of 100 nM Lat B. *EF*, gene for elongation factor ef1α (internal standard); *Pa1-4*, *Picea abies *actin isoforms 1-4. The four columns of bands in each treatment represent samples taken after 3, 5, 7 and 10 days of maturation.

Expression of the four actin isoforms was further analyzed in the non-fractionated ESM on day 3, 5, 7 and 10 of maturation on control media or media with the addition of 100 nM Lat B. In the controls, transcription of isoforms *Pa1 *and *Pa2 *slightly decreased, while transcription of isoforms *Pa3 *and *Pa4 *did not change significantly. In the presence of Lat B, in contrast, transcription of the suspensor-specific actin isoforms *Pa2*, *Pa3 *and *Pa4 *showed a distinct, gradual decline, while the transcript level of *Pa1 *remained more or less unchanged (Figure 5).

### Phylogenetic analysis reveals that gymnosperm and angiosperm actins form distinct sub-families

**Figure 6:**
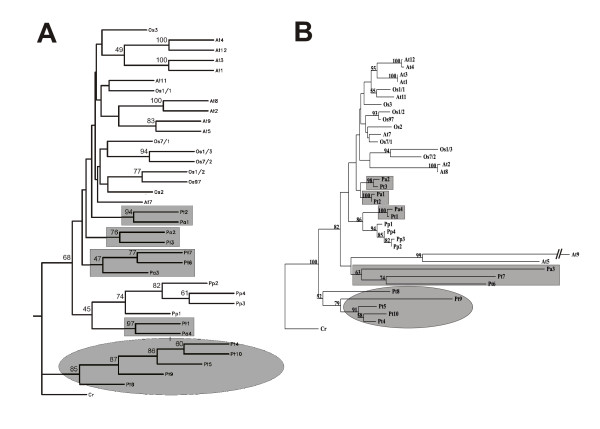
**Rooted phylogenetic trees of actin genes**. The tree was constructed by (A) the maximal likelihood method using Phylip software and (B) the neighbour joining method using Treecon software. Pairs of orthologous genes from Norway spruce AFO 541 (*Picea abies*) and *Pinus taeda *are highlighted in grey rectangles. The grey ellipse highlights a branch of *Pinus taeda *genes with no orthologues isolated from the Norway spruce AFO 541. At, *Arabidopsis thaliana; *Os, *Oryza sativa; *Pa, *Picea abies; *Pp, *Physcomitrella patens; *Pt, *Pinus taeda*; Cr, *Chlamydomonas reinhardtii*. Actin isoform numbers match the gene annotations in the case of *Arabidopsis thaliana *and *Oryza sativa*; isoform labelling in other species is novel.

The protein sequences of the four new actin isoforms from the Norway spruce (*Picea abies*) line AFO 541 were compared with representative actin sequences from another gymnosperm, *Pinus taeda*, the angiosperms *Arabidopsis thaliana *and *Oryza sativa*, and the moss *Physcomitrella patens *(Additional file 1) using phylogenetic analysis (Figure 6). The phylogenetic tree revealed that all four newly identified Norway spruce genes formed pairs of orthologues with genes from the phylogenetically most closely related species *Pinus taeda *(grey rectangles in Figure 6). Surprisingly, the pair of Norway spruce isoform *Pa4 *and pine isoform *Pt1 *clustered in a branch together with actins from the moss *Physcomitrella patens*. The above-mentioned gymnosperm and moss actins clustered together with actins of the angiosperms *Arabidopsis thaliana *and *Oryza sativa*, whereas the rest of *Pinus taeda *isoforms (*Pt4, 5, 8, 9 *and *10*) formed a clearly separated branch (grey oval in Figure 6). No orthologues of these genes were isolated in our experiments with the Norway spruce line AFO 541 despite repeated attempts using either specific or degenerated primers designed according to the *Pinus taeda *isoforms.

### Actin isoform *Pa*3 differs in the sequence of Latrunculin B-binding domain

**Figure 7:**
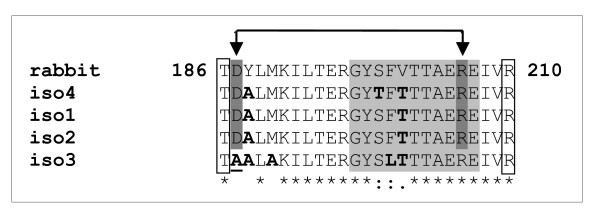
**Multiple alignments of latrunculin-binding region of a rabbit actin and four spruce actin isoforms**. Threonin T186 and arginin R210 directly binding latrunculin are framed with rectangles; aspartic acid D187 and arginin R206 forming a salt bridge (indicated by arrows) are highlighted in dark gray; a loop moving under Lat B binding is in light gray, amino acid exchanges between the rabbit actin sequence and spruce isoforms are in bold. The amino-acid exchange D187→A187 in isoform 3, hypothetically responsible for altered latrunculin binding, is underlined.

In an effort to identify the source of the apparent high sensitivity of the suspensor actin filaments to Lat B, we compared predicted amino acid sequences of suspensor-specific isoforms *Pa2*, *Pa3 *and *Pa4 *with that of the latrunculin-binding region (from T186 to R210) of rabbit actin [19]. Although the amino acids directly responsible for the interaction with latrunculin, threonin T186 and arginin R210, are apparently conserved in all four Norway spruce isoforms, a few differences were detected in the intervening region in the *Pa*3 and *Pa*4 isoforms (Figure 7). Most of the differences represent substitutions of amino acids with similar chemical properties, such as, methionin→alanin, serine→threonin, or phenylalanin→leucin. However, the substitution of aspartic acid D187 to alanin in *Pa*3 necessarily disabled the formation of the salt bridge between the aspartate D187 and arginin R206 (Figure 7), which is important for stabilization of the tertiary structure of actin and Lat B binding [19].

## Discussion

### Expression of actin isoforms is regulated during somatic embryo development

Actin is encoded by large gene families in both metazoans and plants. In plants the number of family members reaches dozens in soybean, tobacco, potato, rice or longpole pine [5,6,20] or even more than 100 members in petunia [21]. In *Arabidopsis*, the actin family consists of eight genes and two pseudogenes. Phylogenetic analyses of actin isoforms in *Arabidopsis *have indicated their diversification into two clusters that are preferentially expressed in either vegetative or generative organs [6,7,9]. Although the causality between the expression pattern and sequence similarity remains unclear, it is apparent that most individual isoforms are not functionally equivalent. The differences in actin sequences are responsible for the characteristic features of either monomeric actin or actin filaments, including interactions with other proteins. Individual actin genes have probably evolved along with other interacting proteins in specific tissues and organs, and therefore their sequence features are tightly interconnected with specific expression levels and spatio-temporal patterns. This was clearly demonstrated by introducing the generative *ACT 1 *gene under the control of the vegetative *ACT2 *promoter into transgenic *Arabidopsis*, which resulted in a significantly increased pool of G-actin, reduced organ size, delayed flowering, and caused dwarfism and other phenotypic changes [9]. Pairs of the most related actin genes in *Arabidopsis *(e.g. *ACT1 *and *3*, *ACT2 *and *8*, *ACT4 *and *12*) encode identical proteins or proteins that differ in only a single amino-acid residue. In spite of these tiny structural differences, these genes have been maintained in the genome for 30-60 millions years [6,7], indicating that their functional differences and specific expression patterns are regulated independently for the two genes in each pair.

No detailed data are available either in *Arabidopsis *or any other plant species about differential expression of individual actin isoforms during embryo development that would be comparable with, for example, the documented changes in actin expression during pollen maturation and germination [22,23]. In *Arabidopsis*, actin genes ACT1, ACT3 and ACT11 were found to be related to zygotic embryogenesis [7]. However, the expression patterns of these "reproductive actin" genes are not limited to embryos because ACT1 and ACT3 are also expressed in ovules, mature pollen and organ primordia, and ACT11 is also expressed in pollen, carpel, seeds and siliques. Most likely the specific expression pattern of actin isoforms is related to specific interactions with actin-associated regulatory proteins in the respective tissues.

In our study of actin functions in somatic embryogenesis in the Norway spruce line AFO 541 we succeeded to identify four new actin genes. Considering the generally high number of actin genes in plants, and almost twice as many actin genes that we have found in the sequence database of *Pinus taeda*, we assume that the four genes do not represent the full spectrum of actins in spruce. Our phylogenetic analysis of actin sequences from *Arabidopsis thaliana*, *Oryza sativa*, *Pinus taeda*, *Physcomitrella patens *and the four newly identified spruce isoforms (Figure 6) revealed that the four isoforms cluster together with angiosperm actins and those from *Physcomitrella patens*. The cluster of *Pinus taeda *isoforms Pt4, 5, 8, 9 and 10 probably represents a more ancient group of actins that might be preferentially expressed in vegetative organs.

The expression of the spruce embryonic actin isoforms was to some extent developmentally regulated. Our results clearly indicated that the expression of *Pa2*, *Pa3 *and *Pa4 *predominated in suspensor cells when compared with meristematic cells, whereas *Pa1 *was expressed more or less equally in both cell types. Differential expression of the four actin isoforms in the suspensor cells may be related to their unique developmental program based on the necessity of rapid polar growth and programmed cell death fate, contrasting with the continuous formative cell divisions in the meristematic centres.

### Latrunculin B preferentially blocks suspensor development

The effects of key actin depolymerizing drugs on somatic embryogenesis of *Picea abies *were studied by Binarová *et al*. [24]. The authors described a relatively high resistance of the thick actin filaments in suspensor cells towards cytochalasin B. Baluška *et al*. [25] showed that treatment with 0.2-5 μM Lat B impaired cell elongation, which resulted in the formation of short suspensors and barrel-shaped late somatic embryos, but the development of the embryos was reported to occur normally. Smertenko *et al*. [18] investigated the effect of either 100 nM Lat B or 5 μM cytochalasin D on the maturation of spruce embryogenic lines. The application of both drugs resulted in gradual destruction of actin cytoskeleton in all cell types, and either caused a reversion of the cultures back to a proembryonic phenotype or was completely lethal [18]. We observed similar devastating effects of Lat B concentrations above 200 nM, which almost completely arrested the maturation process. In contrast, however, 50-100 nM Lat B did not negatively affect the development of high-quality embryos, it only selectively inhibited the formation and growth of suspensors and initiated or accelerated their gradual destruction as shown by cytological analysis (Figure 3). Moreover, the expression of suspensor-specific actin isoforms markedly decreased during the maturation on media supplemented with Lat B, further indicating preferential degradation of the suspensor cells. In contrast to the effect of low concentrations of Lat B, application of cytochalasin D at any concentration either exhibited no cytological/morphological effect (concentrations 0.05 - 0.1 μM) or resulted in a gradual non-specific disintegration and death of both suspensor and meristematic cells (concentrations 0.5 - 2 μM). A study on more detailed description of the morphoregulatory effect of these two anti-actin drugs on spruce AFO 451 SE line and several fir SE lines is in preparation (Vondráková *et al*., unpublished results).

A question arises why the Lat B effect was selectively destructive towards the suspensor cells. The mode of action of Lat B is based on binding to actin monomers, thus preventing their polymerization [19]. In contrast, cytochalasin D blocks the formation of actin filaments through binding to their plus ends [26]. Considering that Lat B binds directly to actin monomers, we assume that the interaction between Lat B and actin may be isoform-specific. This explanation is consistent with our finding that one of the suspensor-specific actin isoforms, *Pa*3, contains an amino-acid substitution D187A, which prevents the formation of a salt bridge important for the interaction with Lat B [19]. Altered affinity of *Pa*3 for Lat B may influence the actin composition in F-actin pool and the dynamics of polymerisation. Such changes may lead to the accelerated death of suspensor cells, which is generally regarded as a prerequisite for normal maturation of both somatic and zygotic embryos [27,28]. In summary, the selective degradation of suspensor cells probably resulted primarily from their ontogenetic predetermination to PCD that was further accelerated by either altered actin dynamics or specific effects related to the altered interaction of *Pa*3 isoform with Lat B. An alternative explanation based on non-specific alteration of actin dynamics or different permeability of Lat B into the two cell types cannot be excluded.

The ability of Lat B to induce PCD in the SE conifer lines has been previously demonstrated by Smertenko *et al*. [18] and further studied by Bozhkov *et al*. [2]. In both studies the role of apoptosis-like mechanisms was demonstrated. Thomas *et al*. [3] convincingly documented the ability of Lat B to induce apoptosis-like PCD associated with caspase-3-like activity in *Papaver rhoeas *pollen grains. In our experiments, the detection of PCD-related DNA fragmentation using either TUNEL test or electrophoretic detection of DNA laddering in developing embryos was negative.

### Degradation of suspensors in early phases of maturation promotes faster development of high-quality embryos

Early somatic embryos in the proliferating Norway spruce line AFO 541 developed in an asynchronous manner. Subsequently, upon transfer to maturation media, embryos in various developmental stages commenced the process of maturation. Only embryos in advanced developmental stages were capable of further development to mature cotyledonary embryos in the four weeks, whereas all other embryos were malformed or arrested in their maturation. As Lat B induced selective degradation of suspensors in embryos at all developmental stages, somatic embryos in the earliest developmental stages containing only small meristematic centres were incapable of further development without functional suspensors and died together with the suspensor cells. In contrast, degradation of suspensors accelerated the development of embryos at later developmental stages. The elimination of the earliest embryos under Lat B treatment resulted in a lower total yield. However, embryos of high quality were preferentially formed at the end of maturation, fully capable of further development. Lat B treatment may thus replace manual sorting of mature embryos, which is a limiting factor in mechanized production of artificial seed. Our results thus provide not only academic, but potentially also practical benefit concerning application of Lat B in conifer micropropagation.

## Conclusions

Our results demonstrated that somatic embryo development is accompanied with spatio-temporally regulated expression of at least four actin genes. Actin cytoskeleton in the two main cell types of the embryogenic cultures (meristematic and suspensor cells) differed in architecture and sensitivity to actin depolymerising drug Lat B. Lat B treatment led to preferential block of suspensor development. The degradation of suspensors in early phases of the maturation accelerated the development of high-quality embryos and caused the disintegration of less developed ones.

## Methods

Embryogenic culture of *Picea abies *L. (Karst.), genotype AFO 541 (AFOCEL, France) was used in all experiments and cultured as follows.

### Somatic embryogenesis

#### Proliferation phase cultivation conditions

The culture was grown on GD media according to Gupta and Durzan [29], solidified with 0.75% agar (Sigma-Aldrich, Steinheim, Germany). The pH was adjusted to 5.8 prior to autoclaving. This medium was supplemented with 0.21 mM cefotaxime (Sefotak, Valeant Czech Pharma, Prague, Czech Republic), 5 μM 2,4-dichlorophenoxyacetic acid (2,4-D), 2 μM kinetin, 2 μM 6-benzylamino purine (BAP) and 30 g/l sucrose (Duchefa, Haarlem, The Netherlands). All phytohormones and organic components except sucrose were diluted separately. The solutions were filter-sterilised and added to cooled autoclaved media. The embryogenic cultures were maintained by weekly subculturing into Magenta vessels (Magenta corporation, Chicago, Illinois) containing 40 ml of fresh medium. Cultures were kept in darkness at 24 ± 1°C.

#### Maturation phase cultivation conditions

In order to initiate the maturation phase, the cytokinins and auxin in the liquid GD medium were substituted with 20 μM abscisic acid (ABA, Sigma-Aldrich) and 3.75% polyethylene glycol 4000 (PEG, Sigma-Aldrich). The PEG solution was autoclaved separately and added to the medium after autoclaving. During maturation the cultures were subcultured twice onto fresh medium of the same composition in Magenta vessels with membrane rafts (Osmotek, Rehovot, Israel). The subcultivating interval was 1 week. Cultures were kept in darkness at 24 ± 1°C for 4 weeks.

#### Desiccation

After four weeks of maturation, fully developed embryos were selected and desiccated. The embryos were carefully transferred onto dry paper in small Petri dishes (3 cm in diameter). These open dishes were placed into large Petri dishes (18 cm in diameter) with several paper layers wetted with sterile water (100% humidity). The dishes were covered with lids, sealed with parafilm, and kept under a light regime of 12 hours of light (PPFD approximately 15-25 μmol m^-2 ^s^-1^, daylight fluorescent tubes; Osram, Wintherthur, Switzerland) and 12 hours of darkness, at 20 ± 1°C for 3 weeks.

#### Germination

Desiccated embryos were transferred into Magenta vessels filled with 40 ml of 1/4 strength GD medium without phytohormones but supplemented with 0.5% sucrose and 0.4% active charcoal (Duchefa), pH 5.8. Dishes with embryos were placed in a cultivation room with light regime of 12 hours of light (PPFD approximately 50 μmol m^-2 ^s^-1^, daylight fluorescent tubes; Osram) and 12 hours of darkness, at 24 ± 1°C for 2 weeks.

### Evaluation of embryogenesis

The structure and appearance of the embryogenic suspensor mass (ESM) was evaluated during the proliferation and maturation phases. Changes induced by the transfer of the ESM from proliferation to maturation media and to media supplemented with latrunculin B (Lat B) were recorded after one and two weeks. The ESM material was placed on a microscopic slide, mixed with one drop of 0.04% trypan blue (Sigma-Aldrich), a cover glass was placed onto the ESM after 2 minutes of exposure, and the dye was rinsed out with distilled water. The preparations were examined using Zeiss Jenaval transmission light microscope. For observation of later stages of maturation, we used Nikon SMZ 1500 stereomicroscope. Images of four-week-old somatic embryos and emblings after two weeks of germination were recorded using Nikon DS-5M digital camera. All images were processed using the computer image analysis system Lucia G, version 5 (Laboratory Imaging, Prague, Czech Republic).

The yield of embryos after four weeks of maturation was evaluated using 10 clusters of embryogenic culture in each variant. Each cluster was spread in 2 drops of distilled water in Petri dish (5 cm) and the image recorded using Nikon DS-5M digital camera. All images were processed using the Lucia G analysis system as above, and the total number of cotyledonary embryos and the length of each embryo were measured.

For quantification of Lat B effect, the length of the embryo at the end of maturation was used as a simple substitutional criterion of embryo maturity. Embryo maturity, however, is defined by complex factors including the size, morphology, biochemical markers or embryo ability to germinate [30]. Generally, embryos longer than 2.5 mm were considered as matured embryos not arrested in their development.

### Latrunculin B application

Latrunculin B (Lat B; Sigma-Aldrich) was dissolved in DMSO (Duchefa) in concentration 2.53 mM (stock solution). Lat B from the stock solution was added into the proliferation or maturation media to achieve final concentrations of 1, 10, 25, 50, 100 and 200 nM. The maximum concentration of DMSO in the medium supplemented with Lat B was 0.01% (v/v). Untreated cultures and those treated with 0.01% (v/v) DMSO were used as controls.

### Viability assay

Cell viability was assessed using fluorescein diacetate (FDA, Sigma-Aldrich) staining according to the method of Widholm [31]. Stock solution of FDA in acetone (2 mg/ml) was freshly diluted with water to 0.02% (w/v) concentration, and 20 μl of this solution were mixed on a microscope slide with ESM resuspended in culture medium.

### Actin visualisation

Actin was visualised using rhodamine-phalloidin staining according to the method of Blancaflor [32]. ESM was incubated for 30 min in PME buffer (50 mM Pipes, 4 mM MgSO_4_.7H_2_O, 10 mM EGTA, Sigma-Aldrich), pH 6.9, supplemented with 300 μM 3-maleimidobenzoyl-*N-*hydroxy-succinimide ester (MBS, Sigma-Aldrich). The samples were then incubated for 10 min in PME supplemented with 0.1 μM rhodamine-phaloidin (Sigma-Aldrich), 0.3 M mannitol (Duchefa) and 2% glycerol (v/v) (Lachner, Neratovice, Czech Republic). Subsequently, the ESM was washed twice for 10 min with PME buffer, mounted on microscope slides and observed immediately under a confocal laser microscope.

### Microscopy

A Zeiss LSM 5 Duo confocal laser-scanning microscope was used for visualization of FDA staining (excitation at 488 nm by Argon/2 laser, emission filter set LP 505). Rhodamine-phalloidin was observed either with the Zeiss 5 LSM Duo (excitation at 561 nm by DPSS laser, emission filter set LP 575) or with Leica TCS SP2 confocal laser scanning microscope (excitation at 543 nm, emission at 545 - 590 nm).

### Isolation and expression analysis of actin isoforms

Total RNA was isolated using Qiagen Plant RNA extraction Kit (Qiagen, Hilden, Germany) from 100 mg of the total ESM cultured for 3, 5, 7, 10 or 14 days on the maturation medium with or without Lat B, or alternatively from embryos isolated from embryogenic culture cultivated for 14 days on the maturation medium. The total RNA was used for reverse transcription using oligo-T_23 _primer and RevertAid™ M-MuLV Reverse Transcriptase (Fermentas, St.Leon-Rot, Germany) according to the manufacturer's instructions. The resulting cDNA was used as a template for subsequent PCR using recombinant *Taq *DNA Polymerase (Fermentas), 1× PCR buffer with 20 mM (NH_4_)_2_SO_4_, 1.5 mM MgCl_2_, 0.2 mM dNTPs and 0.2 μM degenerate primers that were designed to match consensus sequences of conserved domains of actin genes. Primers *ActF*: (GGCATCATACATTCTACAATGAGT) and *ActR1*: (CAACIACITTGATTTTCATGCTGCT) were used for the isolation of isoforms 1 and 2, primers *ActF2*:(ATGGAIAAIATITGGCATCA) and *ActR2 *(ATCCAIACAIIGTAITTCCT) were used for the isolation of isoform 3. Primers for the isolation of isoform 4 were designed according to the specific region of *Pinus taeda *actin isoform 1 (*ActPt1F*: CATTGATGAAGATTCTTACAGAAC, *ActPt1R*: TATTAAAGATGGCTGGAACAGC). Missing 5' ends of isolated segments of actin genes were amplified using isoform-specific reverse primers (see bellow) and forward primers designed according to sequences of related actin isoforms from *Pinus taeda *(*Act1,2ATG*: GAAGATGGCTGACGCWGAGG and *Act4ATG*: ATGGGTGATGCAGAAGAAATCC); missing 3' ends were obtained using isoform-specific forward primers (see bellow) and oligoT_23 _primer or using SMART™ RACE cDNA Amplification Kit and PowerScript™ ReverseTranscriptase (Clontech laboratories, Palo Alto, CA, USA). In case of *Pa3 *isoform, the isolation of missing ends was not successful by any of the methods.

To analyze the expression of individual isoforms, specific primers were designed according to the most variable regions of the four actin genes to allow their specific amplification as follows: *Pa1F*: ACATCCTGTACTTCTTACTGAA and *Pa1R*: CCTGTTCATAGTCCAGTGCC; *Pa2F*: CACCCTATTCTTCTCACTGAG and *Pa2R*: GCTCGTAACTCTTCTCCAGG; *Pa3F*: CAACCGCGAGAAGACGATCG and *Pa3R*: TAGTCCAGCGCCACGTAG; *Pa4F*: AGAGCGCGAGATTGTCCG and *Pa4R*: ACTTCTGCACATCTGAACCGT.

As an internal standard, the transcript for the elongation factor ef1α was used [33] with universal primers generally applicable for amplification of *ef1α *from almost any plant species: *EF1F*: TACTGCACTGTGATTGATGCC [34] and *PtEF1R*: CATCCATCTTGTTACAGCAGC, which was designed to match the sequence of *ef1α *from *Pinus taeda*. The semiquantitative RT-PCR analysis had at least two (usually three) repetitions with similar results for every analysed sample and gene combination.

### Sequence alignments and phylogenetic analysis

Sequences of actin isoforms isolated from *Picea abies *were aligned with sequences found in gene databases using ClustalX [35]. A phylogenetic tree from full length protein sequences (if available) was constructed using either neighbor-joining (NJ) method as implemented in the Treecon software [36] with 1000 bootstrap samples and Poisson correction for distance calculation, or the heuristic approximation of the maximum likelihood (ML) method [37] provided by Proml tool in combination with the Seqboot and Consense tools from the PHYLIP package [38,39]. In ML analysis, the JTT (default) substitution model for amino acids was used.

## Authors' contributions

KS, LH and JF carried out the actin visualization studies. LF designed primers for isolation of actin genes and carried out sequence alignments and phylogenetic analysis. PB and EB isolated the actin isoforms and made the expression analysis. PB was first to describe the positive effect of latrunculin B on the maturation of high quality somatic embryos. KS and KE performed the confocal microscopy studies. ZV carried out majority of tissue culture work and computer image analysis. ZO conceived of the study, and together with MV, KS and LF participated in design and coordination of the study. ZO, KS and LF drafted the manuscript. All authors read and approved the final manuscript.

## Supplementary Material

Additional file 1 List of sequences used for the construction of the phylogenetic tree. Click here for file
